# Comparative Effects of Repeated Linear Sprint and Change-of-Direction Speed Training on Performance, Perceived Exertion and Enjoyment in Youth Soccer Players

**DOI:** 10.3390/sports14010033

**Published:** 2026-01-08

**Authors:** Okba Selmi, Mohamed Amine Rahmoune, Hamza Marzouki, Bilel Cherni, Anissa Bouassida, Antonella Muscella, Santo Marsigliante, Jolita Vveinhardt, Wafa Douzi

**Affiliations:** 1 High Institute of Sports and Physical Education of Kef, University of Jendouba, Kef 7100, Tunisia; okbaselmii@yahoo.fr (O.S.); hamzic_30@hotmail.com (H.M.); bilelcherny@gmail.com (B.C.); bouassida-anissa@yahoo.fr (A.B.); 2 Research Unit, Sportive Performance and Physical Rehabilitation, University of Jendouba, Jendouba 8189, Tunisia; 3 High Institute of Sports and Physical Education of Sfax, University of Sfax, Sfax 3000, Tunisia; amine.iyad2040@gmail.com; 4 Department of Biological and Environmental Sciences and Technologies (Di.S.Te.B.A.), University of Salento, 73100 Lecce, Italy; santo.marsigliante@unisalento.it; 5 Klaipėdos Valstybinė Kolegija/Higher Education Institution, 91274 Klaipeda, Lithuania; jolita.vveinhardt@vdu.it; 6 Laboratory «Mobilité, Vieillissement, Exercice» (MOVE)—UR 20296, Faculty of Sports Sciences, University of Poitiers, 8 Allée Jean Monnet, 86000 Poitiers, France; wafa.douzi01@univ-poitiers.fr

**Keywords:** conditioning, change of direction, adolescent development, intermittent exercise, psychological engagement

## Abstract

Youth soccer requires an integrated approach combining technical–tactical, physical, and psychological components to enhance performance and long-term engagement. Although Repeated Linear Sprint Training (LRST) and Repeated Change of Direction Speed (RCOD) training are widely used to improve fitness, direct comparisons of their effects on physical performance and perceptual responses in adolescent players remain limited. This study compared the effects of an 8-week LRST versus RCOD training program on physical performance, perceived exertion, and enjoyment in youth soccer players. Twenty-six male players were randomly assigned to an LRST group (n = 13) or an RCOD group (n = 13). Both groups completed two weekly sessions of their assigned training in addition to regular soccer practice. Pre- and post-intervention assessments included acceleration and sprint speed, change-of-direction (COD) performance (T-Half Test [THT], Illinois Agility Test [IAT]), lower-limb power (Five-Jump Test [5JT], Squat Jump [SJ], Countermovement Jump [CMJ]), and endurance-intensive fitness. Enjoyment and session-RPE were recorded after each training session. Both groups improved across all physical measures (main effect of time, *p* < 0.0001). Significant time × group interactions favored RCOD for THT (~1.6%), IAT (~1.1%), 5JT (~2.3%), CMJ (~5.2%), and SJ (~6.3%), with no overall main effect of group. Enjoyment was consistently higher in the RCOD group (*p* < 0.0001), while session-RPE did not differ between groups. In youth soccer, both LRST and RCOD effectively enhance physical performance. However, RCOD appears more effective for improving pre-planned COD and explosive performance while eliciting greater enjoyment without increasing perceived exertion. Incorporating structured RCOD training alongside linear sprint work may represent a practical strategy to optimize physical development and sustain player engagement.

## 1. Introduction

Modern soccer places substantial physical and psychological demands on players, requiring a blend of speed, agility, endurance, and cognitive skills such as focus, motivation, and resilience [1]. During matches, athletes must perform high-speed sprints, sharp changes of direction (COD, defined here as pre-planned directional changes), and precise ball control under pressure, while integrating perceptual skills, tactical awareness, and rapid decision-making [2]. These demands underscore the need for holistic training that addresses both physical conditioning and sport-specific performance factors [3,4].

Soccer’s intermittent and multidirectional nature, with frequent accelerations, decelerations, and directional changes, makes developing linear speed, COD ability, and agility (defined here as a reactive ability in response to external stimuli) crucial [5]. Two commonly used training modalities are Repeated Linear Sprint (LRST, corresponding to the classical repeated sprint ability [RSA]) and Repeated Change-of-Direction Speed (RCOD) training. LRST uses repeated straight-line sprints to target anaerobic energy systems and recovery between efforts [6,7], whereas RCOD incorporates repeated short sprints with directional changes, increasing demands on deceleration, re-acceleration, and neuromuscular control [2,8]. Both LRST and RCOD offer complementary benefits. LRST enhances peak linear sprinting and the capacity to sustain repeated high-intensity efforts [9,10], primarily stimulating anaerobic glycolytic pathways and phosphocreatine resynthesis to improve sprint recovery and fatigue tolerance [11]. In contrast, RCOD imposes greater neuromuscular and mechanical strain, challenging sensorimotor integration, proprioceptive feedback, and executive control, thereby supporting rapid pre-planned COD performance rather than reactive agility [2,3,8,12].

LRST session may become monotonous due to the repetitive nature of linear sprints [13,14], whereas RCOD exposes players to frequent high-intensity decelerations and cutting actions that increase mechanical loading and may elevate injury risk if not appropriately progressed [15,16]. Considering these factors, including player enjoyment and perceptual responses in comparative studies is crucial to understanding protocol adherence and feasibility in youth soccer. From a theoretical perspective, enjoyment and sustained engagement can be framed through Self-Determination Theory and flow theory, which emphasize autonomy, competence, and optimal challenge as key drivers of intrinsic motivation and engagement [17,18,19]. RCOD training, with its multidirectional and varied structure, may enhance these psychological experiences compared to the more repetitive LRST, potentially increasing intrinsic motivation and sustained participation.

Despite extensive research on each modality individually, head-to-head comparisons in youth soccer remain limited, especially regarding their effects on both physical performance and perceptual responses such as enjoyment and perceived exertion, which are key determinants of training adherence and long-term participation [20,21,22]. Enjoyable training experiences may be especially important during adolescence, a period of significant neurocognitive development and increased sensitivity to motivational contexts, as they support sustained engagement, effort, and continued sports participation [20,21]. By integrating greater coordinative and motivational involvement, RCOD drills may enhance enjoyment and adherence by supporting the basic psychological needs described in Self-Determination Theory.

To inform youth coaching practice, this study directly compared 8-week LRST and RCOD protocols in Under 17 (U17) male players, examining effects on physical performance, perceived exertion, and enjoyment. We hypothesized that both protocols would lead to significant improvements in physical performance. However, given its emphasis on multidirectional movements and cognitive-perceptual engagement, we further hypothesized that the RCOD protocol would yield greater improvements in COD performance. Additionally, we expected that players in the RCOD group would report higher levels of physical enjoyment, potentially reflecting the greater cognitive, perceptual, and motivational stimulation inherent to RCOD drills.

## 2. Materials and Methods

### 2.1. Participants

Before recruiting participants, a sample size calculation was performed using G*Power software (version 3.1.9.4, University of Kiel, Kiel, Germany) based on a repeated-measures ANOVA design with within-between interaction [23]. The effect size (f = 0.4) was selected based on Cohen’s conventions for a large effect [24]. The analysis indicated that a minimum of 16 participants (effect size f = 0.40, statistical power = 84.5%) was required to detect significant differences, assuming a Type I error rate of 0.05 and a Type II error rate of 0.20 (statistical power = 80%). To account for potential dropout, 26 male youth soccer players (U17 category) from the same club. Participants were classified according to their playing position (central defender, full-back, attacking midfielder, and forward), with goalkeepers excluded due to their distinct training regimens.

Inclusion criteria were: (a) a minimum of six years of soccer experience and regular participation in club training routines; (b) absence of severe musculoskeletal injuries in the past year; and (c) no mild to moderate injuries in the past month. To be included in the final analysis, participants were required to complete at least 90% of all training and testing sessions. All included participants demonstrated high adherence, with mean attendance rates of 95 ± 3% in the LRST group and 96 ± 2% in the RCOD group, confirming the feasibility of implementing these training protocols in a real-world competitive setting.

In order to reduce any bias associated with position-specific physical demands, player positions were carefully considered during randomization, making sure that each group (LRST and RCOD) had an equal representation of positions. Following classification, players were randomly assigned to the LRST (n = 13; age = 15.8 ± 0.4 years, peak height velocity (PHV) = 0.2 ± 0.2 years, height = 175.5 ± 2.8 cm, body mass = 69.8 ± 4.6 kg, and BMI = 22.7 ± 1.0 kg·m^−2^) or RCOD (n = 13; age = 15.3 ± 0.5 years, PHV = 0.0 ± 0.3 years, height = 174.9 ± 3.8 cm, body mass = 68.6 ± 5.5 kg, and body mass index (BMI) = 22.4 ± 1.1 kg·m^−2^) groups using a coin toss. This process guarantees that variations in positional roles are unlikely to confuse observed effects. Allocation concealment was ensured by a blinded assessor who was not involved in the randomization process.

**Figure 1:**
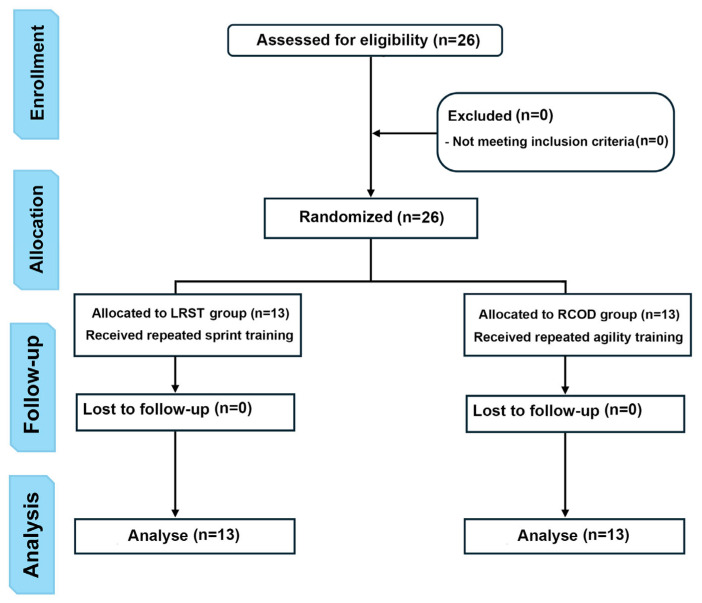
CONSORT (Consolidated standards of reporting trials) Flow diagram of the progress through the phases of a randomized trial of two groups.

All participants competed in the same competitive category (U17; highest level for their age) and followed a standardized weekly microcycle consisting of five training sessions (Tuesday–Saturday; ~40–90 min each) plus one official match on Sunday, ensuring comparable training history and competitive exposure. As shown in Table 1, during the first three training days (Tuesday–Thursday), ~60% of session time was devoted to technical–tactical tasks and ~40% to physical training (aerobic work, small-sided games, and power/anaerobic training). During the final two training days (Friday and Saturday), ~75% of total time was allocated to technical-tactical content (technical drills and simulated competitive games), with the remainder devoted to physical training (speed and reaction-speed work) [25]. This routine was identical for both groups; therefore, the only between-group difference was the conditioning component (RCOD vs. LRSA). Figure 1 presents the CONSORT flow diagram of the study.

Prior to participation, informed consent was obtained from the players’ parents or legal guardians, as all participants were minors. The research protocol was approved by the institutional ethics committee of University of Jendouba, Kef (approval number: 013/2023; approval date: 19 October 2023) and adhered to the Declaration of Helsinki ethical standards.

### 2.2. Experimental Design

This study adopted a repeated-measures design with randomized allocation to the training interventions. The intervention was carried out during the 2023–2024 competitive season (January to March), lasting 10 weeks, which comprised two testing weeks (pre- and post-intervention) and eight weeks of specific training. Subjects were assigned to either the LRST or RCOD protocols, with both interventions matched for weekly and total training volume and performed in addition to the players’ regular soccer training regimen. Given the athletes’ elite competitive level, the in-season timing of the intervention, the proximity of official matches and international tournaments, and the limited flexibility in training planning, participants were allocated to two active training groups (LRST and RCOD), and no regular-training-only control group was included. During this competitive phase, all players were required to follow performance-oriented conditioning strategies integrated within the team’s preparation, making the implementation of an additional control condition impractical. This approach allowed for an ecologically valid comparison between two applied training modalities under real-world competitive constraints.

At both assessment points (pre- and post-intervention), participants underwent a battery of physical performance tests including sprint speed (10 m and 30 m sprints), COD (T-half agility test and Illinois Agility Test [IAT]), vertical jump (squat jump [SJ] and countermovement jump [CMJ]), horizontal jump (five-jump test [5JT]), and endurance-intensive (Yo-Yo Intermittent Recovery Test Level 1 [YYIRT1]). The same evaluators, who were blinded to group allocation, conducted all assessments. Testing sessions were scheduled approximately 48 h after the participants’ last competition or high-intensity training session to minimize the fatigue effect. All tests were conducted under standardized conditions (e.g., on a synthetic grass soccer field) with verbal encouragement, across two non-consecutive evenings (Tuesday and Thursday) between 17:00 and 19:00. The first session focused on acceleration, speed and COD tests, while the second assessed jumping and endurance-intensive performance. Each session was preceded by a standardized 15 min warm-up including jogging, active stretching, and dynamic exercises (e.g., short sprints, skipping). Except the Yo-Yo test, which was performed once, all other tests involved two valid maximal trials separated by two minutes of passive recovery. The best performance was retained for analysis. A five-minute passive recovery period was provided between different tests.

One week before the intervention, all participants completed two familiarization sessions (~48 h apart) to ensure technical proficiency in the testing and training procedures, including the use of the Borg Rating of Perceived Exertion (RPE; 1–10 scale) [26]. and the Physical Activity Enjoyment Scale (PACES) [2,27]. During the familiarization week, anthropometric assessments were also conducted. Body mass was recorded to the nearest 0.1 kg using a digital scale (OHAUS, Florham Park, NJ, USA), height was measured to the nearest 0.01 m, and BMI was calculated by dividing body mass (kg) by the square of height (m^2^). Biological maturity was determined using the Mirwald et al. [28] method, which calculates a “maturity offset” from age, leg length, sitting height, and body weight. Based on this offset from their predicted PHV, participants were categorized as Pre-PHV (−3 to >−1 years), Circa-PHV (−1 to +1 years), or Post-PHV (>+1 to +3 years). All players were classified as Circa-PHV. The reliability of the physical tests (i.e., intraclass correlation coefficient (ICC_3,1_)), Coefficient of variation [CV]) was calculated using the results from the familiarization sessions and those obtained during the pre-test.

The training interventions were integrated into the players’ regular weekly schedules, with two additional sessions per week dedicated to either the LRST or RCOD protocols. Training sessions were closely monitored to ensure protocol adherence and consistency. To monitor training load and subjective responses, participants completed a training journal twice weekly after each LRST or RCOD session. Training stimulus was systematically monitored even though GPS and heart rate (HR) data were not collected. This was done by (i) making sure that the LRST and RCOD sessions matched for weekly and total training volume, (ii) recording session-RPE (sRPE) and PACES after each training session, and (iii) having the same research team supervise all sessions to ensure methodological consistency. All training and testing sessions were supervised by the same research team to ensure methodological consistency across procedures. To further standardize testing conditions, participants were instructed to refrain from intense physical activity on the day preceding evaluations. They were also encouraged to maintain consistent sleep patterns and regular training routines throughout the study to minimize confounding.

### 2.3. Procedures

#### 2.3.1. Acceleration and Speed Assessment

To assess acceleration and speed performance, participants performed the 10 and 30m linear sprint tests at maximal effort, respectively. Sprint time (seconds) was recorded using a series of paired photocells (Globus, Microgate, Bolzano, Italy). The photocells were placed at a 0.2 m height at the start line, with a front-foot positioned at 0.5 m behind this point, and at a 1 m height at the 10 m and 30 m marks [5]. The ICCs for S10 and S30 were 0.910 and 0.900, respectively, and their corresponding CVs were 1.13% and 0.54%.

#### 2.3.2. Change of Direction Assessment

The IAT was used due to its high reliability and relevance for evaluating an athlete’s ability to accelerate, decelerate, turn, and change direction quickly [29]. The test was performed at maximal effort along a standardized course layout as previously described [29]. Sprint time (in seconds) was recorded using a series of paired photocells (Globus, Microgate, Bolzano, Italy). The photocells were positioned at a 0.2 m height at the starting line, with a front-foot marker placed 0.5 m behind this point, and at a height of 1 m at the finish line. ICC and CV of IAT were 0.900 and 0.62%, respectively.

The THT was used as a valid and reliable measure of COD in team sport athletes, incorporating forward, lateral, and backward movements in a shorter, more compact course [8]. The test was performed in an all-out mode and conducted according to a strict course layout, as described by Haj-Sassi et al. [8]. The total completion time (in seconds) was measured using timing gates (Globus, Microgate, Bolzano, Italy). ICC and CV of THT were 0.890 and 0.98%, respectively.

#### 2.3.3. Jumping Assessment

The SJ was used as it provides a reliable measure of pure lower-body explosive strength without the influence of the stretch-shortening cycle [30]. Participants began in a static squat position with knees flexed at approximately 90°, hands placed on the hips, and without any preparatory movement. After holding the position for 2–3 s, they performed a vertical jump as high as possible. Moreover, the CMJ was used as a valid and reliable measure of lower-limb power and the efficiency of the stretch-shortening cycle [30]. Participants started from a standing position, then performed a rapid downward movement (eccentric phase), followed immediately by a vertical jump (concentric phase), keeping their hands on their hips throughout. Jump height (in cm) was measured using an infrared jump system for both tests (Optojump; Microgate, Bolzano, Italy). The ICC was 0.880 for both tests, with CVs of 1.62% for SJ and 1.81% for CMJ.

The 5JT was used as a reliable and practical test for evaluating lower-limb explosive power and horizontal jump performance in youth and adult athletes [31]. It involves five consecutive strides, starting from a standing position with both feet on the ground. The athlete performs five maximal forward jumps by alternating legs and finishes by landing on both feet. The total horizontal distance covered (meter), from the starting line to the landing point, was measured in meters. ICC and CV of the test were 0.994 and 0.74%, respectively.

#### 2.3.4. Endurance-Intensive Fitness Assessment

The YYIRT1 was employed as a validated and reliable tool for assessing athletes’ endurance-intensive performance [32]. It consists of repeated 20 m shuttle runs at progressively increasing speeds, interspersed with 10 s of active recovery intervals. The test continues until the participant fails twice to reach the line on time. The highest velocity reached in the last completed stage was used to estimate maximal aerobic speed (MAS), while the total distance covered was used to estimate maximal oxygen consumption (VO2max) [32]. The maximal oxygen consumption (VO2max) was estimated from the total distance covered in the YYIRT1 using the predictive equation proposed by Bangsbo et al. [32]:

VO2max (mL·kg^−1^·min^−1^) = 0.0084 × total distance (m) + 36.4

Intrasubject reliability and intersession reliability were determined during familiarization sessions, showing excellent consistency: ICC = 0.92 and CV = 5.8%.

#### 2.3.5. Rating of Perceived Exertion

Three minutes after each specific training, participants rated their exertion level using Borg’s 10-point Likert scale (0–10 AU) [26], with 0 corresponding to ‘no exertion’ and 10 to ‘maximal exertion’.

#### 2.3.6. Physical Enjoyment Assessment

The 8-item PACES assessed participants’ enjoyment of physical activity by asking them to rate their feelings about a recent session on a 7-point scale for each item, where 1 means “I hate it” and 7 means “I like it” [27]. The total score, ranging from 8 to 56, was calculated by summing individual item ratings, with higher scores indicating greater enjoyment. Participants completed the questionnaire two minutes after each specific training session. The PACES questionnaire was completed directly following the cool-down phase, using a paper-and-pencil format.

The items of the PACES questionnaire were translated into the native language of the participants, by a panel of three professional translators with proven expertise in psychological scale translation. Each translator independently translated all items, and the consistency across versions was evaluated. For each item, agreement among the translators was scored as 1 when all versions matched, and as 0 in the case of any discrepancies. Overall agreement across all items was then analyzed, yielding an inter-translator consistency coefficient of 0.88. This high level of agreement reflects the quality and accuracy of the translation process, confirming that the adaptation of the PACES preserved the original intent and semantic integrity of the items.

#### 2.3.7. Training Protocols

Over the eight-week training program, participants completed either LRST or RCOD trainings immediately after the standardized warm-up at the beginning of regular team sessions. The warm-up included jogging, dynamic stretching, jumps, and sprints with and without changes in direction. A three-minute rest interval separated the warm-up from the start of the main exercise.

During the first four weeks, each session consisted of two blocks of six maximal-intensity repetitions to ensure consistent effort. Each repetition was followed by twenty seconds of rest, with a three-minute rest between blocks to allow partial recovery and restoration of performance. To promote continued adaptation, training volume was progressively increased during the final four weeks by adding a third block of six repetitions, while maintaining the same rest intervals. This adjustment adhered to the principles of progressive overload and recovery. Both protocols were conducted at the same time (18:30 h) and on the same days (Tuesdays and Thursdays) to ensure standardized training conditions. Exercises, distances, and rest intervals are detailed in Table 2.

The LRST protocol was designed to enhance sprint performance and anaerobic endurance. It involved six maximal-intensity 20 m straight-line sprints [33], simulating the repeated high-intensity efforts commonly encountered in sports like soccer.

**Figure 2:**
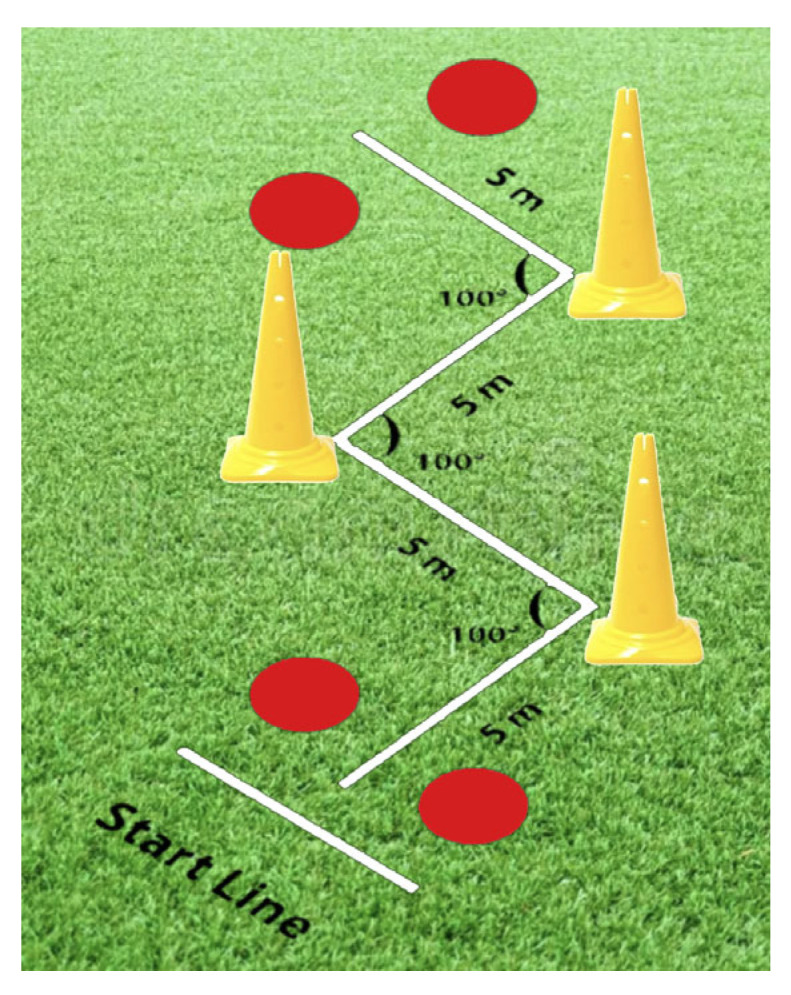
Diagram for Zigzag agility drill.

The RCOD protocol was adapted from the zigzag agility drill described by Little and Williams [34], consisting of four 5 m segments connected by 100° angles, requiring three sharp changes in direction over a 20 m course (see Figure 2). This design effectively stimulates the multidirectional agility demands of team sports, emphasizing key components such as deceleration, rotation, and rapid reacceleration.

### 2.4. Statistical Analyses

All data are expressed as mean ± standard deviation (SD). To assess whether the variables followed a normal distribution, the Shapiro–Wilk test was performed, while Levene’s test was used to evaluate homogeneity. Independent sample t-test was conducted to compare demographic and anthropometric attributes. The intrasubject reliability (individual variability across training sessions) and intersession reliability (group variability across training sessions) of the training load and enjoyment responses were calculated for each group and expressed as the coefficient of variation (CV%) with confidence interval (95% CI), using the spreadsheet developed by Hopkins [35]. CV% values ≤ 12% were considered acceptable [36]. For internal-load and enjoyment scores (normality not assumed), the Mann–Whitney U test was used for comparisons between distinct groups. Pearson’s r was employed to estimate the magnitude of significant findings as effect size for internal-load and enjoyment scores, with the thresholds defined as follows: small = 0.3, medium = 0.5, large = 0.6 [37]. A two-way analysis of variance (ANOVA) with repeated measures (2 Times × 2 Groups) was used to examine the main and interaction effects across all normally distributed variables. For post hoc multiple comparisons, adjustments for family-wise error were applied using the Bonferroni or Games-Howell tests, depending on variance homogeneity. Given the significant baseline differences in the THT, a two-way analysis of covariance (ANCOVA) using the pre-test scores as a covariate to compare groups was conducted. Effect sizes for ANOVA and ANCOVA were reported as partial eta-squared (*η*^2^*p*) and interpreted as follows: small (0.01 < *η*^2^*p* < 0.06), medium (0.06 ≤ *η*^2^*p* < 0.14), and large (*η*^2^*p* ≥ 0.14) [38]. Cohen’s d was calculated to quantify the magnitude of the differences within and between groups, and classified as follows: small = 0.1, medium = 0.4, large = 0.8 [37]. The percentage change from pre- to post-test (∆) was calculated for all measures. Statistical analyses were conducted using SPSS version 26 for Windows (IBM Corp, Armonk, NY, USA), with the significance level set at *p* ≤ 0.05.

## 3. Results

No significant differences were observed between groups across all training sessions in RPE scores. However, intrasubject PACES scores were significantly less variable during RCOD training compared to LRST (Table 3). Significant differences in PACES scores were found between groups across all training sessions, with higher scores (*p* < 0.0001) in RCOD group (Table 3).

Except for T-half agility test (*p* = 0.028; d = 0.92), RCOD group showed no significant differences in age, PHV, anthropometrics and physical variables compared to LRSA group at baseline (all *p* > 0.05) (Table 4 and Table 5).

A significant main effect of time was observed for all physical variables (all *p* < 0.0001), with both groups significantly improving their performance after the intervention period (*p* < 0.0001; Table 4 and Table 5). Large time × group interactions were observed only for THT, IAT, 5JT, CMJ, and SJ (all *p* < 0.05). For THT and IAT, RCOD players were 1.6% and 1.1% faster than LRST. For 5JT, CMJ, and SJ, RCOD players were 2.3%, 5.2%, and 6.3% more explosive than LRST, respectively. No main effect of group was found for any physical variable (*p* > 0.05; Table 4 and Table 5).

## 4. Discussion

The current study aimed to directly compare the effects of LRST and RCOD training on physical performance, internal-load responses, and enjoyment in young male soccer players. The findings revealed that both training methods significantly enhanced physical performance across multiple measures. Large, significant interactions were observed for THT, IAT, 5JT, CMJ, and SJ, with RCOD showing potentially faster and more explosive performance improvements than LRST. The RCOD protocol also elicited higher enjoyment, which may be related to its multidirectional and cognitively engaging nature.

Across outcomes, both groups demonstrated meaningful improvements in acceleration/speed, COD, jumping, and intensive-endurance outcomes, supporting the efficacy of high-intensity intermittent training for improving sport-specific physical capacities in youth athletes. These findings align with previous literature suggesting that repeated linear sprint and COD-based training enhance anaerobic and aerobic fitness [5,7,39]. However, where time × group interactions emerged (THT, IAT, 5JT, CMJ, SJ), the pattern favored RCOD, suggesting that planned multidirectional braking and propulsion more directly target the neuromuscular qualities underpinning pre-planned COD performance and explosive actions, rather than reactive agility. These observations align with recent syntheses showing that COD-oriented and plyometric stimuli meaningfully enhance COD and jump ability in youth/adolescent soccer populations [40,41].

The significant time × group interactions observed for THT and IAT indicate greater gains in planned COD speed following RCOD than LRSA. Mechanistically, RCOD drills involve repeated eccentric braking, rapid re-orientation, and explosive re-acceleration within pre-defined movement patterns, which may enhance eccentric braking capacity, intermuscular coordination, and lower-limb stiffness/force transmission [42]. These adaptations could plausibly contribute to faster performance in non-reactive COD tests such as the IAT and THT, which primarily assess planned COD speed rather than perceptual–decision components [42].

The significant interactions for 5JT, CMJ, and SJ may reflect preferential neuromuscular adaptations associated with RCOD training. Frequent eccentric-concentric transitions and stretch-shortening-cycle (SSC) loading inherent to planned directional changes could potentially underpin the larger RCOD-related gains in horizontal (5JT) and vertical (CMJ, SJ) explosive outputs, consistent with meta-analytic evidence in adolescent soccer [40].

Taken together, the interaction effects support a specificity-driven account grounded in pre-planned multidirectional braking/propulsion cycles. By repeatedly overloading eccentric rate control, coordination, and SSC efficiency, which may be enhanced within fixed patterns, could potentially develop the capacities required by the THT, IAT, 5JT, CMJ, and SJ tests, providing a plausible explanation for the group-specific gains. These inferences are biomechanically plausible and supported by contemporary reviews of COD determinants and neuromuscular adaptations in football [16,42,43,44]. Importantly, the present findings pertain to pre-planned agility only and should not be extrapolated to reactive agility paradigms.

By contrast, sprint performance (e.g., S10, S30) improved similarly over time in both groups, with no significant interaction effects observed. These results suggest that, while high-intensity field work generally benefits linear speed, RCOD training did not provide additional advantages over LRST for purely linear sprint outcomes. From a specificity perspective, repeated linear sprint training would be expected to preferentially enhance short-distance sprint performance. The absence of a clear advantage for LRST in this study may reflect insufficient training volume or intensity of the linear sprint sessions, or interference/moderation from concurrent team-soccer training, which could have attenuated modality-specific adaptations. These findings are consistent with previous youth soccer studies, where COD-focused and linear sprint training produced comparable improvements in sprint and pre-planned COD performance when weekly exposure was low (e.g., one to two sessions) [45,46,47,48,49]. This highlights the importance of training dose and contextual factors when interpreting specificity-driven adaptations.

Participants in the RCOD group reported consistently higher PACES scores across sessions than LRST, despite the drills being pre-planned rather than reactive. This pattern likely reflects the greater movement variety and multidirectional demands that reduce perceived monotony [50,51], the frequent, salient performance feedback from executing fixed-angle cuts at prescribed speeds, which supports perceived competence and autonomy (core drivers of intrinsic motivation in Self-Determination Theory) [52], and a stimulating yet tolerable internal-load profile typical of well-designed high-intensity field work in youth soccer [2]. Notably, the enjoyment advantage emerged without any reactive or decision-making components, indicating that the structured characteristics of COD drills, rather than cognitive novelty, underpin the motivational response [2]. Practically, embedding COD blocks within youth microcycles may help sustain motivation and adherence [53,54] while concurrently targeting the pre-planned agility and jump adaptations observed in our time × group interactions.

Additionally, despite the higher enjoyment observed with RCOD, RPE scores did not differ between groups across the intervention. The sRPE was statistically equivalent, suggesting that the greater motivational response to RCOD was achieved without increasing the athletes’ perceived internal load. This supports the practicality of substituting RCOD blocks for some linear sprint work when the goal is to enhance engagement while keeping perceived effort comparable.

For youth programs seeking to improve agility and explosive power while maintaining enjoyment, we suggest incorporating pre-planned COD blocks (e.g., fixed-angle cuts, zigzags, 505/T patterns) about twice per week for 6–8 weeks, alongside LRST or resisted sprints to support linear speed. Practitioners and coaches can rotate a small set of pre-planned drills to limit monotony, set clear yet achievable technical targets (such as plant angle, controlled deceleration, and split times), and provide brief, task-focused feedback. It can be helpful to monitor perceived exertion, and enjoyment together (e.g., sRPE plus periodic PACES). If enjoyment declines, practitioners and coaches can make modest adjustments to sprint volume, drill spacing or angles, or drill selection, while maintaining pre-planned patterns and respecting the actual work-to-rest ratio of the protocol. A light mid-block reduction in load may also support both adaptation and motivation.

This study presents several limitations that should be acknowledged. First, the small sample size limits statistical power and generalizability of the findings. Second, the inclusion of only male participants restricts the applicability of the results to female athletes. Third, the relatively short intervention period may have been insufficient to capture long-term adaptations or retention effects. Without follow-up data, the durability of the observed adaptations remains unknown. While the RCOD involves multidirectional movements, no direct cognitive performance assessments (e.g., reaction time, decision-making) were included to support potential cognitive involvement, limiting our understanding of the interplay between physical and psychological adaptations, particularly in agility-focused training. Moreover, the absence of physiological or biomechanical measurements, such as electromyographic activity, metabolic markers (e.g., blood lactate, muscle oxygenation), or tendon stiffness, precludes a detailed mechanistic understanding of the observed adaptations. Although training load was carefully monitored via sRPE, PACES, training logs, and supervision, GPS and HR monitoring were not employed. This limits the objectivity of quantifying mechanical and physiological load (e.g., maximal speed, number of CODs) and should be considered a methodological limitation. Future studies could incorporate these measures to provide a more comprehensive assessment of training load and its effects. In youth populations, some of the observed adaptations may partially reflect natural biological development (growth and maturation), which should be considered when interpreting the effects of the LRST and RCOD interventions.

## 5. Conclusions

Both LRST and RCOD improved key physical capacities in U17 male soccer players over the intervention. RCOD produced comparatively greater gains in COD and jump performance, while linear sprint and endurance-intensive fitness outcomes improved similarly in both groups. Enjoyment was consistently higher with RCOD, yet RPE was comparable between groups, indicating that the motivational benefits of RCOD did not come at the cost of greater perceived exertion. Practically, integrating pre-planned COD blocks alongside linear sprint work appears effective for enhancing pre-planned agility and explosive power while sustaining engagement. These findings should be interpreted in light of the study’s sample and duration, and future work should examine broader cohorts and longer interventions to confirm generalizability and clarify underlying mechanisms.

## Figures and Tables

**Table 1:** Weekly training routine of youth soccer players during intervention.

Day	Main Objective(s)	Time	Duration (min)
Monday	Day off (physical and mental recovery)	-	-
Tuesday	Aerobic training + technical-tactical drills	18:30–20:00	80–90
Wednesday	Small-sided games + technical-tactical drills	18:30–20:00	80–90
Thursday	Power/anaerobic training + technical-tactical drills	18:30–20:00	80–90
Friday	Speed training + technical drills + simulated competitive games	17:00–19:00	80–90
Saturday	Reaction speed + technical-tactical drills	13:00–14:30	40–50
Sunday	Official match	-	-

**Table 2:** Overview of the 8-week training program.

Week	Number of Sessions	RCOD	LRST	RPE (AU)
1	Pre intervention testing			
2–5	8	2 × (6 × 20 m of COD drill at maximum effort); r = 20 s, R = 3 min	2 × (6 × 20 m of linear sprint at maximum effort); r = 20 s; R = 3 min	6–7
6–9	8	3 × (6 × 20 m of COD drill at maximum effort); r = 20 s; R = 3 min	3 × (6 × 20 m of linear sprint at maximum effort); r = 20 s; R = 3 min	6–7
10	Post intervention testing			

RCOD, Repeated Change of Direction Speed training; LRST, Repeated linear Sprint Ability training; RPE, rating of perceived exertion; AU, arbitrary unit; R, rest between bouts; r, rest between repetitions, s, seconds; m, meters; min, minutes.

**Table 3:** Internal-load responses and enjoyment scores of the Repeated Linear Sprint (LRST) and Repeated Change of Direction Speed (RCOD) training groups.

				Variability (CV%)		Group Comparisons		
Variable	Group	Mean ± SD	95% CI	Intersession, 95% CI	Intrasubject, 95% CI	*Z*	*p*	*r*
**RPE (AU)**	LRST	6.7 ± 0.6	6.3–7.1	13.9–15.7	10.5–13.4	0.258	0.797	0.050
	RCOD	6.6 ± 0.6	6.3–7.0	13.7–16.2	10.5–13.4			
**PACES (AU)**	LRST	41.6 ± 3.5	39.4–43.7	8.4–9.1	6.8–10.7	3.462 †	<0.0001	0.679
	RCOD	47.8 ± 3.3	45.8–49.8	7.9–8.8	4.8–5.4			

The values are expressed as mean and SD with 95% confidence interval (95% CI) of the 16 training sessions performed during the intervention period by both RCOD and LRST training groups. RPE: rate of perceived exertion; AU: arbitrary unit; PACES: physical activity enjoyment scale scores; Z: Mann-Whitney U test; *r*: Pearson’s r effect size. The intersession CV% represents the mean variability of the training load responses with the group across the intervention period and expressed as coefficient of variation; intrasubject CV% represents the mean variability of the training load responses and enjoyment scores of the individual subjects across the intervention period and expressed as coefficient of variation. † A significant intergroup difference. The statistical significance level was set at *p* ≤ 0.05.

**Table 4:** Changes in acceleration, speed and change in direction variables in both groups.

Variable	Group	Pre	Post	d (*p*)	Δ (%)	ANOVA/ANCOVA *
**S10 (s)**	LRST	1.98 ± 0.12	1.88 ± 0.08 †	0.981 (<0.0001)	−4.8 ± 4.4	Time: F_1,24_ = 32.274; *p* < 0.0001; *η*^2^*p* = 0.573 Group: F_1,24_ = 1.651; *p* = 0.211; *η*^2^*p* = 0.064 Time × Group: F_1,24_ = 2.516; *p* = 0.126; *η*^2^*p* = 0.095
	RCOD	2.01 ± 0.13	1.96 ± 0.13 †	0.385 (0.008)	−2.7 ± 1.7	
**S30 (s)**	LRST	4.76 ± 0.17	4.65 ± 0.15 †	0.686 (<0.0001)	−2.5 ± 1.2	Time: F_1,24_ = 50.646; *p* < 0.0001; *η*^2^*p* = 0.678 Group: F_1,24_ = 1.364; *p* = 0.254; *η*^2^*p* = 0.054 Time × Group: F_1,24_ = 1.365; *p* = 0.254; *η*^2^*p* = 0.054
	RCOD	4.82 ± 0.19	4.73 ± 0.15 †	0.526 (<0.0001)	−1.7 ± 1.8	
**THT (s)**	LRST	5.88 ± 0.11 ‡	5.81 ± 0.14 †	0.487 (0.002)	−1.2 ± 1.1	Time: F_1,24_ = 56.321; *p* < 0.0001; *η*^2^*p* = 0.701 Group (adjusted for pre-test): F_1,23_ = 3.710; *p* = 0.067; *η*^2^*p* = 0.134 Time × Group: F_1,24_ = 9.426; *p* = 0.005; *η*^2^*p* = 0.282
	RCOD	6.02 ± 0.18	5.85 ± 0.12 †	0.806 (<0.0001)	−2.7 ± 1.5	
**IAT (s)**	LRST	18.15 ± 0.64	18.02 ± 0.59 †	0.211 (0.012)	−0.7 ± 1.0	Time: F_1,24_ = 50.346; *p* < 0.0001; *η*^2^*p* = 0.677 Group: F_1,24_ = 0.741; *p* = 0.398; *η*^2^*p* = 0.029 Time × Group: F_1,24_ = 10.430; *p* = 0.004; *η*^2^*p* = 0.303
	RCOD	18.45 ± 0.56	18.11 ± 0.53 †	0.624 (<0.0001)	−1.8 ± 0.7	

**Note:** Values are expressed as mean ± SD. S10: 10 m sprint; S30: 30 m sprint; THT: T-half agility test; IAT: Illinois agility test; RCOD: Repeated Change of Direction Speed group; LRST: Repeated Linear Sprint training group; d: Cohen’s d; *η*^2^*p*: partial eta squared; Δ: percentage changes; *: ANCOVA was used to test the group effect; † Significant intragroup difference; ‡ Significant intergroup difference at Pre-test. Note: Because the ANCOVA model includes the pre-test THT as a covariate, the residual degrees of freedom are reduced by one (df_error = N—groups—covariates = 26 − 2 − 1 = 23). Statistical significance was set at *p* ≤ 0.05.

**Table 5:** Changes in jumping and endurance-intensive fitness variables in both groups.

Variable	Group	Pre	Post	d (*p*)	Δ (%)	ANOVA
**5JT (m)**	LRST	11.4 ± 0.7	11.6 ± 0.7 †	0.352 (0.001)	2.2 ± 2.3	Time: F_1,24_ = 69.642; *p* < 0.0001; *η*^2^*p* = 0.744 Group: F_1,24_ = 0.912; *p* = 0.349; *η*^2^*p* = 0.037 Time × Group: F_1,24_ = 7.738; *p* = 0.01; *η*^2^*p* = 0.244
	RCOD	11.0 ± 0.8	11.5 ± 0.9 †	0.621 (<0.0001)	4.5 ± 1.8	
**CMJ (cm)**	LRST	35.2 ± 3.0	37.3 ± 2.7 †	0.736 (<0.0001)	6.0 ± 3.8	Time: F_1,24_ = 85.575; *p* < 0.0001; *η*^2^*p* = 0.781 Group: F_1,24_ = 1.413; *p* = 0.246; *η*^2^*p* = 0.056 Time × Group: F_1,24_ = 6.571; *p* = 0.017; *η*^2^*p* = 0.215
	RCOD	33.1 ± 2.7	36.7 ± 3.3 †	1.194 (<0.0001)	11.2 ± 5.8	
**SJ (cm)**	LRST	31.1 ± 2.7	32.4 ± 2.6 †	0.490 (0.002)	4.3 ± 2.8	Time: F_1,24_ = 35.433; *p* < 0.0001; *η*^2^*p* = 0.596 Group: F_1,24_ = 1.269; *p* = 0.271; *η*^2^*p* = 0.050 Time × Group: F_1,24_ = 9.735; *p* = 0.005; *η*^2^*p* = 0.288
	RCOD	29.0 ± 3.1	32.0 ± 3.0 †	0.983 (<0.0001)	10.6 ± 6.1	
**Distance (m)**	LRST	2049.2 ± 307.7	2203.1 ± 283.1 †	0.521 (0.010)	8.1 ± 8.1	Time: F_1,24_ = 23.329; *p* < 0.0001; *η*^2^*p* = 0.491 Group: F_1,24_ = 1.528; *p* = 0.228; *η*^2^*p* = 0.059 Time × Group: F_1,24_ = 0.759; *p* = 0.392; *η*^2^*p* = 0.031
	RCOD	1886.2 ± 283.7	2017.7 ± 261.0 †	0.482 (<0.0001)	12.8 ± 14.2	
**MAV (km·h^−1^)**	LRST	17.0 ± 0.8	17.4 ± 0.8 †	0.500 (0.010)	2.5 ± 2.7	Time: F_1,24_ = 23.102; *p* < 0.0001; *η*^2^*p* = 0.490 Group: F_1,24_ = 1.522; *p* = 0.229; *η*^2^*p* = 0.059 Time × Group: F_1,24_ = 0.738; *p* = 0.399; *η*^2^*p* = 0.029
	RCOD	16.5 ± 0.8	17.1 ± 0.7 †	0.798 (0.001)	3.6 ± 3.8	
**Estimated VO2max (mL·min^−1^·kg^−1^)**	LRST	55.8 ± 2.2	56.9 ± 2.0 †	0.523 (0.010)	2.0 ± 2.2	Time: F_1,24_ = 23.333; *p* < 0.0001; *η*^2^*p* = 0.493 Group: F_1,24_ = 1.530; *p* = 0.228; *η*^2^*p* = 0.060 Time × Group: F_1,24_ = 0.761; *p* = 0.392; *η*^2^*p* = 0.031
	RCOD	54.6 ± 2.0	56.2 ± 1.9 †	0.820 (<0.0001)	3.3 ± 3.3	

The values are expressed as mean and SD. 5JT: five jump test; CMJ: countermovement jump; SJ: squat jump test; MAV: maximal aerobic velocity; VO2max: maximal oxygen consumption; RCOD: Repeated Change of Direction Speed group; LRST: Repeated Linear Sprint group; d: Cohen’s d; *η*^2^*p*: partial eta squared; Δ: percentage changes; † A significant intragroup difference. The statistical significance level was set at *p* ≤ 0.05.

## Data Availability

The original contributions presented in the study are included in the article, further inquiries can be directed to the corresponding author.
